# Cost-effectiveness analysis of sintilimab plus chemotherapy for advanced or metastatic esophageal squamous cell carcinoma

**DOI:** 10.3389/fonc.2022.986762

**Published:** 2022-12-08

**Authors:** Maojin You, Yufan Huang, Zhongjie Cai, Qingfeng Wu, Wei Zhu, Ying He, Ruijia Chen

**Affiliations:** ^1^ Department of Pharmacy, Mindong Hospital Affiliated to Fujian Medical University, Ningde, Fujian, China; ^2^ Department of Emergency Medicine, Mindong Hospital Affiliated to Fujian Medical University, Ningde, Fujian, China; ^3^ Department of Pharmacy, Mengchao Hepatobiliary Hospital of Fujian Medical University, Fuzhou, Fujian, China

**Keywords:** cost-effectiveness, sintilimab plus chemotherapy, esophageal squamous cell carcinoma, first-line treatment, placebo plus chemotherapy

## Abstract

**Background:**

Sintilimab plus chemotherapy (SIDCHM) is more effective than placebo plus chemotherapy (PLCHM) for advanced or metastatic esophageal squamous cell carcinoma (ESCC). However, considering the high cost of sintilimab, this study evaluated the cost-effectiveness of SIDCHM in comparison with PLCHM for advanced or metastatic ESCC from the Chinese healthcare system perspective.

**Methods:**

Polymorphic Markov models were constructed to simulate the course and cost of SIDCHM. Treatment drug costs were calculated at national list prices and clinical data, other costs, and utility values were extracted from the reference literature. Primary outcomes included quality-adjusted life-years (QALYs) and incremental cost-effectiveness ratios (ICERs). The robustness of the model was verified by one-way sensitivity analysis and probabilistic sensitivity analysis (PSA).

**Results:**

SIDCHM obtained 1.03 QALYs at $24,044.49, whereas the effectiveness and cost of PLCHM were 0.67 QALYs and $14,166.24, respectively. The ICER for SIDCHM versus PLCHM was $23,458.08/QALY. The utility of the PFS state was the parameter that had the greatest effect on the ICER. The PSA showed that SIDCHM had an 86% probability of being cost-effective at the willingness-to-pay threshold of 3* Chinese gross domestic product per capita ($37,653/QALY).

**Conclusion:**

From the Chinese healthcare system perspective, SIDCHM is considered a cost-effective treatment option compared with PLCHM as first-line therapy for advanced or metastatic ESCC.

## Introduction

Esophageal cancer (EC) is one of the most frequently occurring malignancies of the digestive tract, ranking seventh in the incidence of malignant tumors worldwide; it is also a very aggressive and lethal disease (1, 2). In the histological subtype, the most common EC is squamous cell carcinoma (2), and the rate of esophageal squamous cell carcinoma (ESCC) is high in China, accounting for more than half of all patients with ESCC worldwide (3). Platinum drugs in combination with fluorouracil or paclitaxel are recommended as a standard first-line therapy currently for advanced or metastatic ESCC (4). In China, platinum plus paclitaxel is generally used, and platinum plus fluorouracil was chosen in preference in other countries (5). However, the median survival of patients with ESCC treated with standard first-line therapy is only 7.0–13.0 months, with very unsatisfactory results (6). The development of new therapy protocols for patients with advanced or metastatic ESCC, as a result, is an urgent matter.

Immune checkpoint inhibitor (ICI) which enhances the antitumor activity of T cells by blocking the programmed cell death protein 1 (PD-1) or cytotoxic T lymphocyte antigen 4 (CTLA-4) pathways, has shown breakthroughs in cancer therapy and has been effective in the treatment of EC in recent years (7, 8). The outcome of the phase II study by Xu et al. showed that the PD-1 inhibitor sintilimab significantly improved the overall survival of patients with advanced or metastatic ESCC after first-line chemotherapy compared to chemotherapy (9). A recent investigational phase III clinical study (ORIENT-15) evaluated the effect of sintilimab or placebo in combined chemotherapy (cisplatin plus paclitaxel or fluorouracil) as first-line therapy for unresectable locally advanced, recurrent, or metastatic ESCC. Sintilimab plus chemotherapy (SIDCHM) showed encouraging results with significant advantages in overall survival (OS, 16.7 vs 12.5 months, P < 0.001) and progression-free survival (PFS, 7.2 vs 5.7 months, P < 0.001) in comparison with placebo plus chemotherapy (PLCHM) (5).

Despite the significant advantages of sintilimab in the treatment of advanced or metastatic ESCC, we cannot ignore its high costs. Therefore, an economic evaluation of SIDCHM as a first-line treatment for advanced or metastatic ESCC based on the ORIENT-15 trial from the Chinese healthcare system perspective was designed.

## Methods

### Model structure

**Figure 1:**
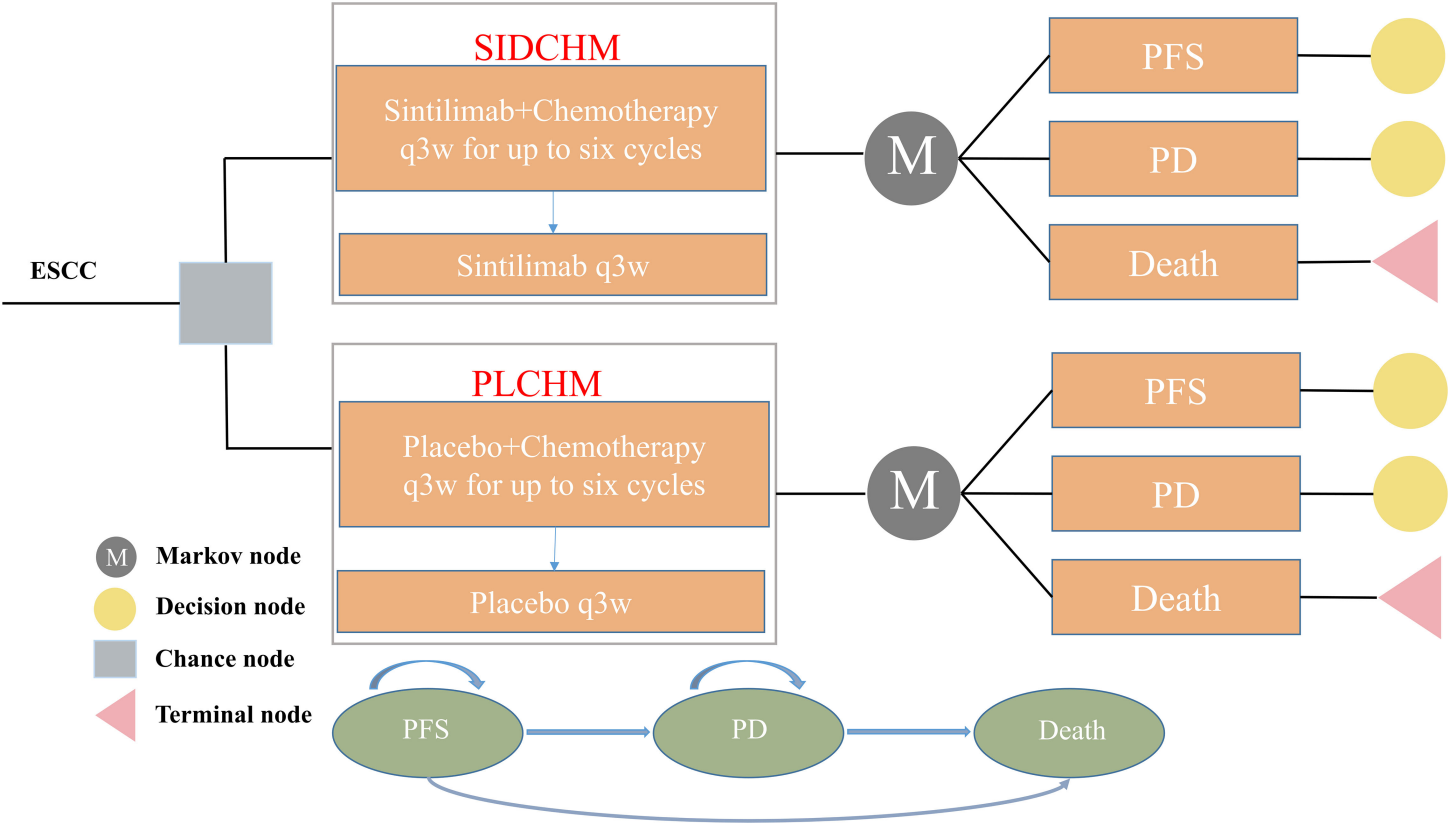
The Markov model simulates outcomes for the ORIENT-15 trial. All patients with ESCC started with PFS state and received treatment with SIDCHM or PLCHM. ESCC, esophageal squamous cell carcinoma; PD, progressive disease; PFS, progression-free survival; PLCHM, placebo plus chemotherapy; SIDCHM, sintilimab plus chemotherapy.

A Markov model was constructed to simulate the cost and effectiveness of SIDCHM, compared with PLCHM, as treatment in the first line for patients with advanced or metastatic ESCC in China. TreeAge Pro 2022 (TreeAge Software, LLC, USA) was used to develop the model and the R software (version 4.2.0) program was used for statistical analyses. Kaplan–Meier survival curves were numerically digitized to select the best-fit survival distribution. Finally, the Weibull survival distribution was used to generate the probability of metastasis for SIDCHM and PLCHM (**Table 1**). The model comprised three mutually exclusive health states: PFS, progressive disease (PD), and death (**Figure 1**) and has a run time of approximately 6 years (to be determined by the time at which 99% of the patients are assumed to die), and each cycle is 21 days long in this model. Through each cycle, patients either hold their assigned health status or advance to new health status and are not allowed to revert to their former health status. The background mortality rate in a Chinese context (10) was also calculated in the model. The output data we eventually had from the model was the total cost, quality-adjusted life-year (QALY), and incremental cost-effectiveness ratio (ICER). We set the willingness-to-pay (WTP) threshold at $37,653 (three times China’s GDP in 2021 per capita), as recommended by the World Health Organization, and consider the treatment option cost-effective if the ICER is below our predefined WTP threshold.

**Table 1:** Relevant parameters of survival distribution.

Parameters	Value	Source
Weibull survival model of PFS		
SIDCHM	Scale = 0.039683, Shape = 1.101677	(5)
PLCHM	Scale = 0.031633, Shape = 1.437746	(5)
Weibull survival model of OS		
SIDCHM	Scale = 0.010568, Shape = 1.284650	(5)
PLCHM	Scale = 0.010993, Shape = 1.4354050	(5)

OS, overall survival; PFS, progression-free survival; PLCHM, placebo plus chemotherapy; SIDCHM, sintilimab plus chemotherapy.

### Clinical data

Data on the clinical efficacy and adverse events were obtained from the ORIENT-15 trial (5). The trial screened patients of ESCC (Their inclusivity criteria were age≥18 years with histologically unresectable locally regionally advanced, recurrent, or metastatic ESCC; unsuitable for curative surgery or definitive concurrent chemoradiotherapy; no prior history of systemic therapy, etc.) and then randomized eligible ones to either the SIDCHM group or the PLCHM group. Sintilimab or placebo was administered per cycle (21 days) until disease progression or unacceptable toxicity developed. Chemotherapy drugs (cisplatin plus paclitaxel or fluorouracil) were also given once every 21 days, that is, cisplatin at 75 mg/m^2^ per cycle, paclitaxel at 175 mg/m ^2^ per cycle, or fluorouracil at 800 mg/m^2^ per cycle. Chemotherapy was provided for up to six cycles; thereafter, treatment was continued with sintilimab or placebo until disease progression or unendurable toxicity. Sintilimab or placebo lasted for a maximum of 24 months. If chemotherapy is not resistible, sintilimab or placebo was provided. Chemotherapy (cisplatin plus paclitaxel or fluorouracil) was not convertible during the study. We assumed that patients of the SIDCHM group received 200 mg of sintilimab and that patients in both arms received the best supportive care (BSC) after disease progression occurs.

### Costs and utilities

We have only calculated direct medical costs, including costs of drugs, tests (e.g., laboratory tests and radiological tests), follow-up, end-of-life care, management of adverse events of severe grade >3, and BSC. Drug costs were based on national tender prices, and other costs were based on published publications. Prices were adjusted to 2021 prices using the China Statistics Bureau Medical Price Index. All costs were expressed in dollars and converted at the average of the 2021 exchange rate (1 dollar = 6.45 RMB). The utility values for PD and PFS were taken from the published literature (11) as there were no relevant quality-of-life data from the ORIENT-15 trial. Costs and utilities were discounted, and the discounted value was 3% per year.

### Sensitivity analysis

To examine the robustness of the model, this study provides a sensitivity analysis of our model, including one-way sensitivity analysis and probabilistic sensitivity analysis (PSA). We adjusted all of the variables up or down within a specified range to establish the most economically influential parameters for the one-way sensitivity analysis. The final results were represented as a Tornado diagram. The maximum and minimum values of these variables were extracted from the literature, and in the presence of missing data the base value of ±20% was used, and the discount rate was used as the lower and upper limits of 0 and 5%, respectively (**Table 2**). The PSA was used to verify the effect of the factors on the uncertainty of the results, and we performed 1000 replications of Monte Carlo simulations with all parameters assigned with the distribution appropriate in the model (**Table 2**). The results were expressed as a probabilistic scatter plot and cost-effectiveness acceptability curves.

**Table 2:** The basic parameters of the input model and the range of sensitivity analyses.

Variables	Base value	Range		Distribution	Source
		Min	Max		
SIDCHM: incidence of AEs					
Anemia	0.125	0.100	0.150	Beta	(5)
Leukopenia	0.174	0.139	0.209	Beta	(5)
Nausea\vomiting	0.043	0.034	0.052	Beta	(5)
Neutropenia	0.300	0.240	0.360	Beta	(5)
Asthenia\decreased appetite	0.040	0.032	0.048	Beta	(5)
Thrombocytopenia	0.028	0.022	0.034	Beta	(5)
Febrile neutropenia	0.024	0.019	0.029	Beta	(5)
PLCHM: incidence of AEs					
Anemia	0.102	0.082	0.122	Beta	(5)
Leukopenia	0.223	0.178	0.268	Beta	(5)
Nausea\vomiting	0.033	0.026	0.040	Beta	(5)
Neutropenia	0.337	0.270	0.404	Beta	(5)
Asthenia\decreased appetite	0.045	0.036	0.054	Beta	(5)
Thrombocytopenia	0.030	0.024	0.036	Beta	(5)
Febrile neutropenia	0.018	0.014	0.022	Beta	(5)
Costs ($)					
Anemia	510.23	408.18	612.28	Gamma	(11)
Leukopenia	467.86	374.29	561.43	Gamma	(11)
Nausea\vomiting	49.42	39.54	59.30	Gamma	(12)
Neutropenia	84.21	67.37	101.05	Gamma	(12)
Asthenia\ decreased appetite	126.84	101.47	152.21	Gamma	(13)
Thrombocytopenia	1058.22	846.58	1269.86	Gamma	(14)
Febrile neutropenia	997.41	797.93	1196.89	Gamma	(15)
Paclitaxel (100 mg)	117.83	94.26	141.40	Gamma	#
Cisplatin (100 mg)	11.63	9.30	13.96	Gamma	#
Fluorouracil (100 mg)	1.86	1.49	2.23	Gamma	#
Sintilimab (100 mg)	167.44	133.95	200.93	Gamma	#
Routine follow-up cost per cycle	73.86	59.09	88.64	Gamma	(11)
Cost of laboratory tests and radiological examinations	358.03	286.42	429.63	Gamma	(11)
Cost of end-of-life care	1831.90	1465.52	2198.28	Gamma	(16)
Cost of best supportive care	167.96	134.37	201.55	Gamma	(11)
Utility value					
PFS	0.68	0.54	0.82	Beta	(11)
PD	0.42	0.34	0.50	Beta	(11)
Proportion					
Receiving cisplatin plus paclitaxel	0.93	0.74	1.12	Beta	(5)
Receiving cisplatin plus fluorouracil	0.07	0.056	0.084	Beta	(5)
Body surface area (m^2^)	1.72	1.38	2.06	Normal	(11)
Discount rate (%)	3	0	5	Fixed	(17)

#, hospital charges; AE, adverse event; PD, progressive disease; PFS, progression-free survival; PLCHM, placebo plus chemotherapy; SIDCHM, sintilimab plus chemotherapy.

### Subgroup analysis

We performed subgroup analyses of all patients by using subgroup-specific hazard ratios reported from the ORIENT-15 trial (5) based on the method of Hoyle et al. (18).

## Results

### Base-case analysis

The base case showed that the SIDCHM group achieved 1.03 QALYs at $24,044.49. The effectiveness was 0.67 QALYs at $14,166.24 in the PLCHM group. The incremental effect and cost of SIDCHM compared with PLCHM were 0.36 QALYs and $9878.25, respectively. The ICER for SIDCHM compared with PLCHM was $23458.08/QALY (**Table 3**). In China, SIDCHM is a cost-effective treatment strategy compared to PLCHM when the cost-effectiveness WTP threshold is $37,653/QALY.

**Table 3:** Effectiveness and costs in the model.

Regimen	PLCHM	SIDCHM	Incremental
Total QALYs	0.67	1.03	0.36
Total cost, $	14166.24	24044.49	9878.25
ICER, $			
Per QALY			23458.08

ICER, incremental cost-effectiveness ratio; PLCHM, placebo plus chemotherapy; QALY, quality-adjusted life year; SIDCHM, sintilimab plus chemotherapy.

### Sensitivity analysis

**Figure 2:**
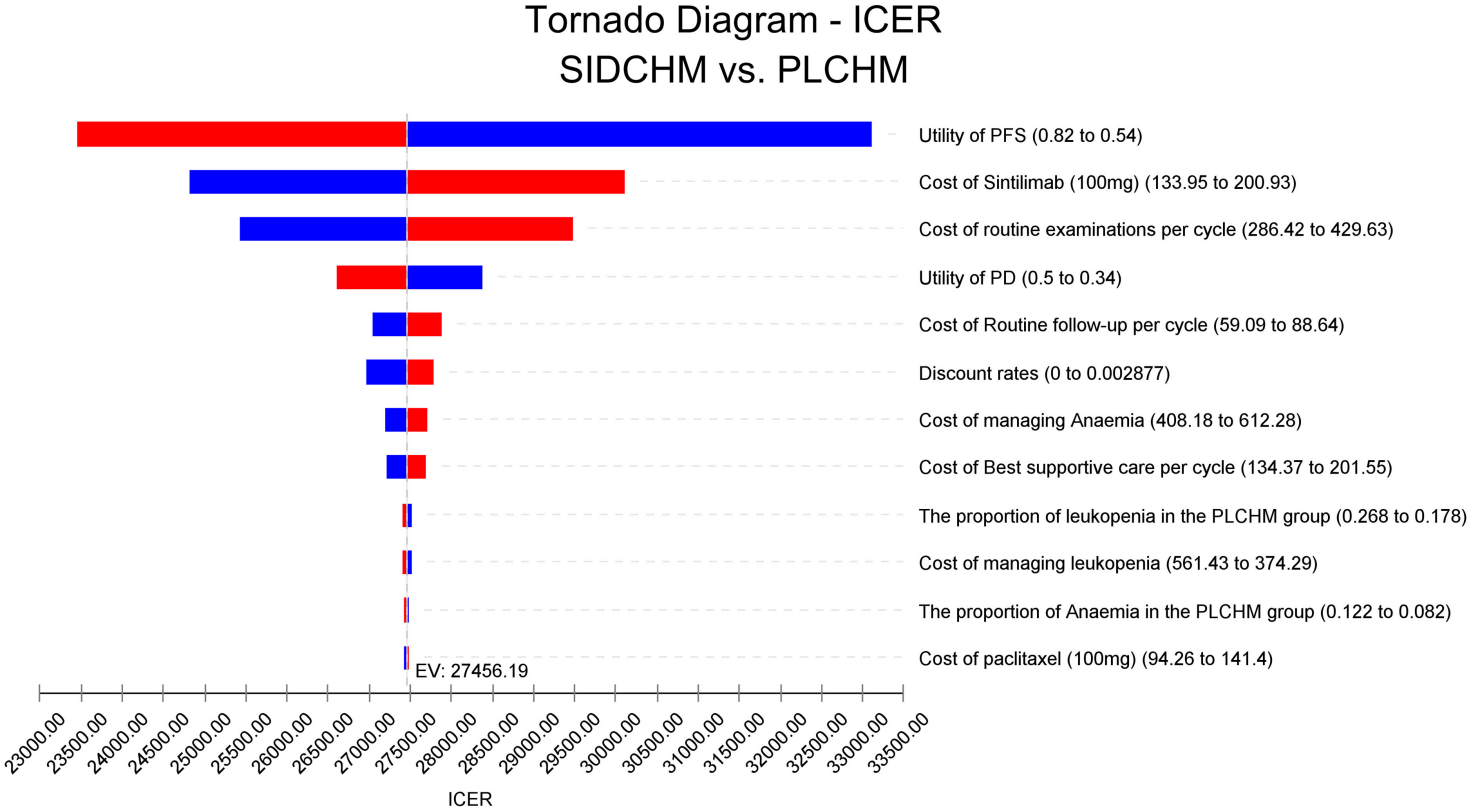
One-way sensitivity analyses of SIDCHM in comparison with PLCHM in China. BSA, body surface area; PD, progressive disease; PFS, progression-free survival; PLCHM, placebo plus chemotherapy; SIDCHM, sintilimab plus chemotherapy7.

**Figure 3:**
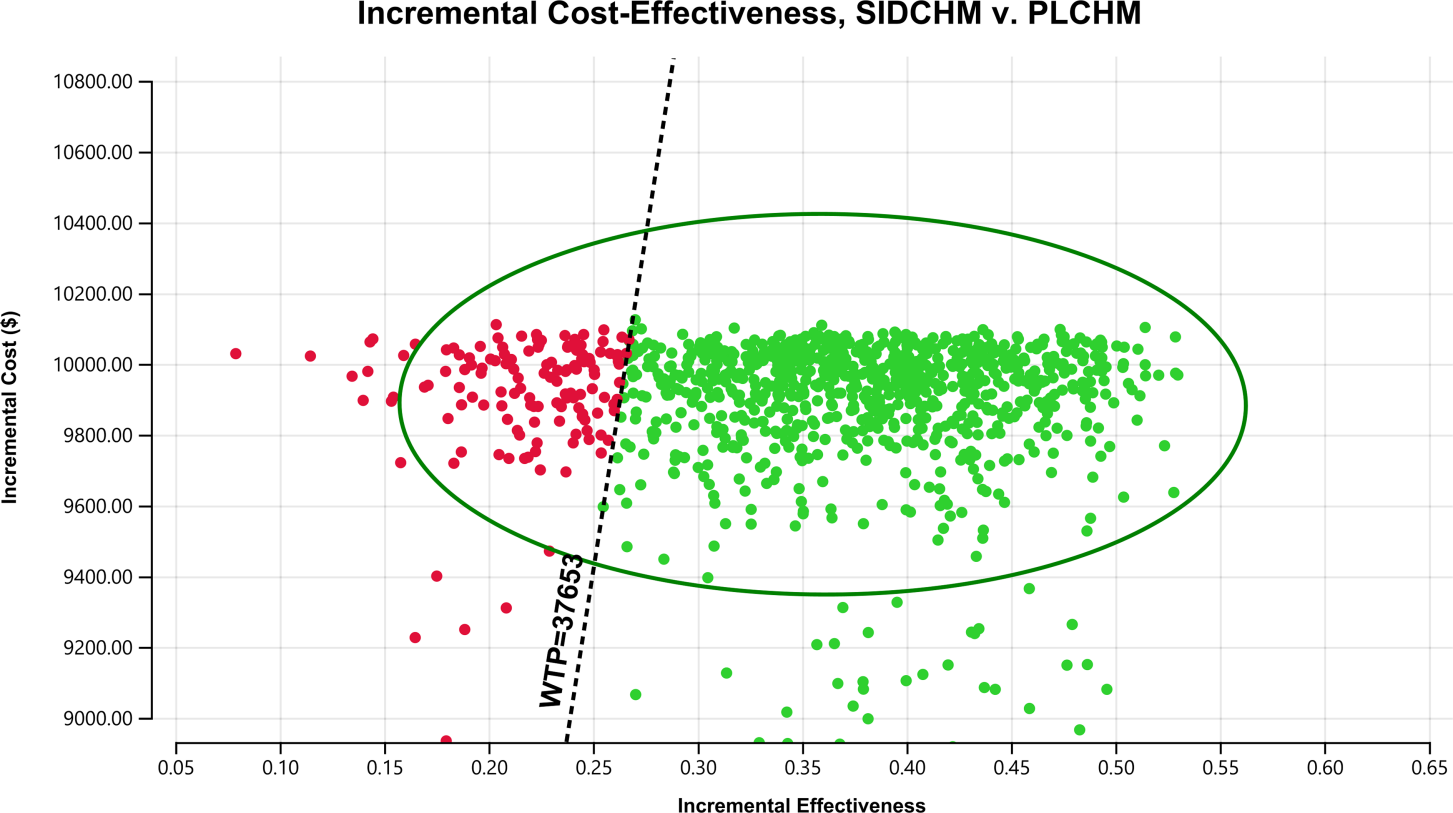
A probabilistic scatter plot of the ICER between the SIDCHM group and the PLCHM group. Each point means the ICER for 1 simulation. Ellipses are used to indicate 95% confidence intervals. Points that lie below the ICER threshold represent cost-effective simulations. ICER, incremental cost-effectiveness ratio; PLCHM, placebo plus chemotherapy; SIDCHM, sintilimab plus chemotherapy; WTP, willingness-to-pay.

**Figure 4:**
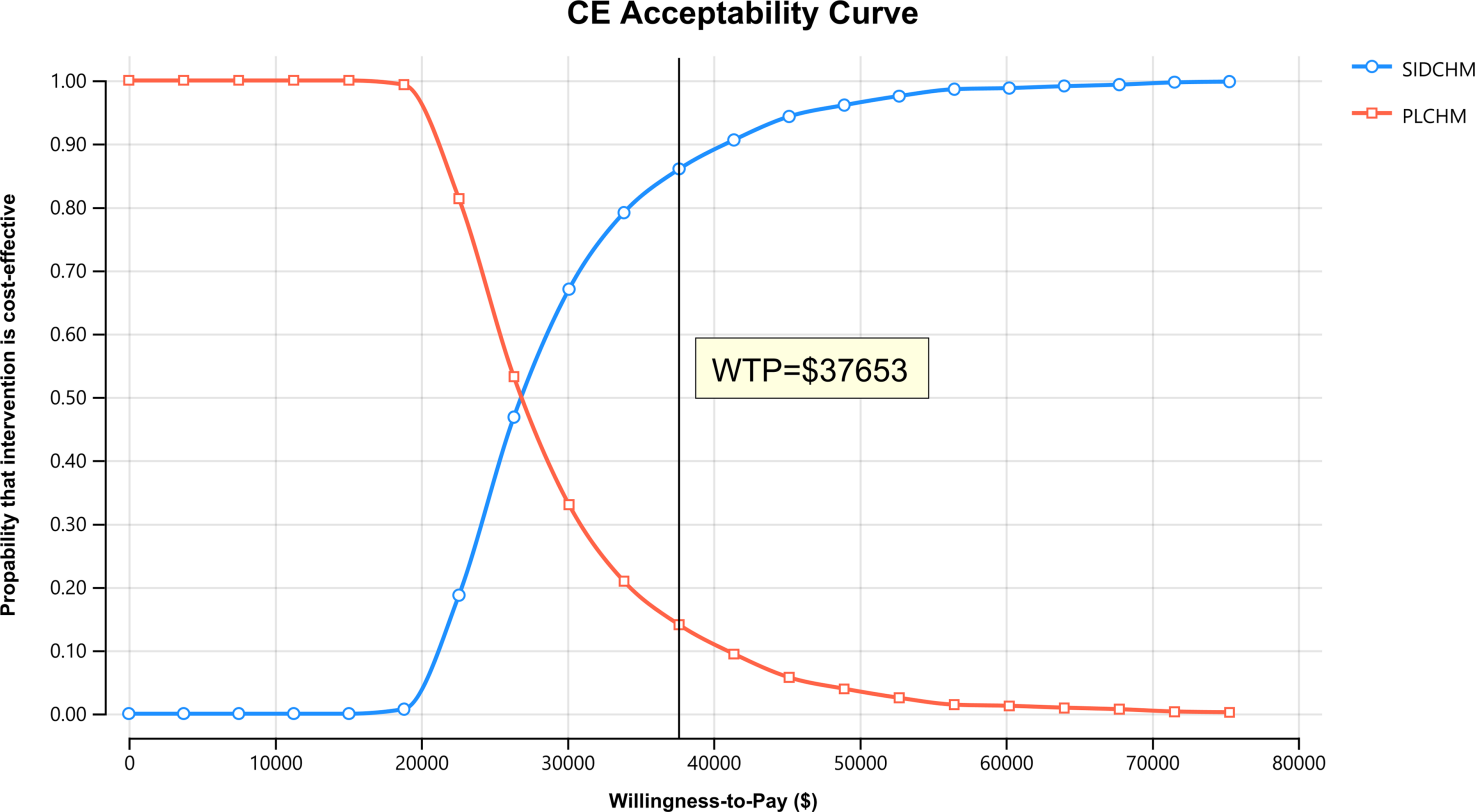
The cost-effectiveness acceptability curves for the SIDCHM treatment option compared to the PLCHM treatment option. PLCHM, placebo plus chemotherapy; QALY, quality-adjusted life year; SIDCHM, sintilimab plus chemotherapy; WTP, willingness-to-pay.

In the one-way sensitivity analysis, results were presented in a Tornado diagram (**Figure 2**). The utility value of PFS and the cost of sintilimab (100 mg) had the most important influences on the results of the model, and the parameters that had relatively minor effects on the model were the cost of laboratory tests and radiological examinations, the utility value of PD, cost of follow-up, etc. However, even if the values of these variables were changed, the ICER was always below our predefined WTP threshold. The results of the PSA are expressed as a probabilistic scatter plot (**Figure 3**) and cost-effectiveness acceptance curve (**Figure 4**), with an 86% probability that the SIDCHM group was cost-effective compared with the PLCHM group when the WTP threshold was $37,653.

### Subgroup analysis

For most subgroups, the ICER of SIDCHM compared with PLCHM was less than the WTP threshold of $37653/QALY, ranging from $26751.55/QALY (probabilities of cost-effectiveness, 87.1%) in patients with age ≥50 years to $335806.56/QALY (probabilities of cost-effectiveness, 53.6%) in patients with Eastern Cooperative Oncology Group performance status =0 (**Table 4**). Only in the subgroup of patients with programmed cell death ligand 1 expression (CPS)<1, the ICER of SIDCHM compared with PLCHM was higher than the WTP threshold of $37653/QALY (**Table 4**).

**Table 4:** Results of subgroup analyses.

Subgroup	OS HR (95% CI)	PFS HR (95% CI)	ICER ($/QALY)	Cost-effectiveness probability (%)
Sex				
Men	0.64 (0.51 to 0.81 )	0.56 (0.46 to 0.69)	32577.34	68.9
Women	0.57 (0.29 to 1.12 )	0.60 (0.34 to 1.07)	33792.10	63.0
Age				
< 65 years	0.70 (0.54 to 0.92 )	0.66 (0.52 to 0.84)	26751.55	87.1
≥ 65 years	0.54 (0.38 to 0.77 )	0.45 (0.32 to 0.62)	28519.93	83.2
Weight(kg)				
< 60	0.67 (0.51 to 0.88 )	0.59 (0.46 to 0.76)	34212.82	64.5
≥ 60	0.59 (0.41 to 0.83 )	0.51 (0.38 to 0.69)	30547.14	74.7
Country or region				
China	0.63 (0.51 to 0.78 )	0.56 (0.46 to 0.68)	32407.35	71.7
ECOG performance status				
0	0.59 (0.36 to 0.96 )	0.68 (0.46 to 1.00)	35806.56	53.6
1	0.64 (0.50 to 0.81 )	0.52 (0.42 to 0.65)	31302.74	75.8
Disease type				
Metastatic	0.62 (0.49 to 0.77 )	0.57 (0.46 to 0.69)	32575.83	68.4
Local advanced	0.77 (0.41 to 1.44 )	0.54 (0.29 to 1.00)	35678.27	58.0
Hepatic metastasis				
No	0.64 (0.50 to 0.82 )	0.54 (0.43 to 0.67)	31975.12	69.9
Yes	0.60 (0.40 to 0.91 )	0.63 (0.44 to 0.91)	34394.85	62.2
Chemotherapy				
cisplatin plus paclitaxel	0.65 (0.52 to 0.80)	0.55 (0.45 to 0.67)	32384.74	71.0
cisplatin plus 5-fluorouracil	0.31 (0.08 to 1.20)	0.55 (0.23 to 1.32)	29955.09	72.5
PD-L1 expression (CPS)				
CPS<10	0.62 (0.45 to 0.85)	0.53 (0.40 to 0.71)	31492.38	70.7
CPS ≥10	0.64 (0.48 to 0.85)	0.58 (0.45 to 0.75)	33326.68	65.3
CPS<5	0.56 (0.37 to 0.86)	0.51 (0.35 to 0.75)	30396.16	73.6
CPS ≥5	0.65 (0.51 to 0.83)	0.58 (0.47 to 0.73)	33428.42	65.3
CPS<1	1.32 (0.63 to 2.77)	0.76 (0.41 to 1.38)	107721.84	25.1
CPS ≥1	0.59 (0.47 to 0.74)	0.54 (0.44 to 0.66)	31270.97	75.6
PD-L1 expression (TPS) (%)				
TPS<10	0.67 (0.52 to 0.88)	0.56 (0.44 to 0.71)	33112.99	68.6
TPS ≥10	0.55 (0.38 to 0.78)	0.54 (0.39 to 0.74)	30951.88	71.7
TPS<5	0.61 (0.46 to 0.82)	0.57 (0.44 to 0.73)	32509.58	68.9
TPS ≥5	0.67 (0.49 to 0.92)	0.54 (0.40 to 0.73)	32524.29	68.7
TPS<1	0.71 (0.53 to 0.95)	0.52 (0.39 to 0.68)	30367.97	78.2
TPS ≥1	0.63 (0.51 to 0.78)	0.59 (0.46 to 0.77)	35057.11	58.7

CPS, combined positive score; ECOG, Eastern Cooperative Oncology Group; HR, hazard ratio; ICER, incremental cost-effectiveness ratio; OS, overall survival; PD-L1, programmed cell death ligand 1; PFS, progression-free survival; QALY, quality-adjusted life years; TPS, tumour proportion score.

## Discussion

In China, EC is the fourth most frequent cause of cancer death and the sixth most prevalent type of cancer, with 300,000 deaths and approximately 320,000 new cases in 2020 (19). ESCC histologically is more prevalent in China, accounting for 90% of all EC cases (20, 21). Palliative chemotherapy as first-line therapy for advanced or metastatic ESCC not only has a restricted survival advantage but also has a poor prognosis and a high number of adverse effects. ICI can significantly improve survival duration and quality of living in a variety of cancers through the inhibition of CTLA-4 or PD-1 pathways (22, 23). ICI plus chemotherapy has become the standard first-line therapeutic option for advanced or metastatic ESCC according to the 2022 guideline for EC treatment (24). Paclitaxel plus cisplatin in combination with camrelizumab is the first-line therapy choice for advanced or metastatic ESCC. The cisplatin plus fluorouracil chemotherapy regimen is combined with pembrolizumab as the first-line treatment for advanced or metastatic EC. Thus far, to the best of our knowledge, only two economic evaluations of camrelizumab or pembrolizumab plus chemotherapy as a first-line treatment option compared with chemotherapy alone for advanced or metastatic EC have been conducted, but neither is cost-effective (11, 25). From a Chinese healthcare system perspective, Zhang et al. concluded that the probability of camrelizumab in addition to chemotherapy being cost-effective as a first-line treatment option for advanced or metastatic ESCC was lower than about 1% when compared with conventional chemotherapy at a cost-effectiveness threshold of $31,498.70, and the factor that had the greatest impact on the ICER was the cost of 200 mg of camrelizumab (11). The results of the economics study by Zhu et al. revealed that the first-line regimen of pembrolizumab plus fluorouracil and cisplatin for EC may not be as cost-effective as fluorouracil and cisplatin from a Chinese economic perspective, and the sensitivity analysis found that the price effect of pembrolizumab was the greatest (25). The high price of ICI will be a major constraint in making it a cost-effective solution. Therefore, searching for an ICI that is less expensive and has good results in treating advanced or metastatic ESCC is essential. Sintilimab is an ICI Self-developed in China and has a significant price advantage over other ICIs imported abroad. More promisingly, in the ORIENT-15 study, Lu et al. used SIDCHM for the first time to treat advanced or metastatic ESCC and showed that compared to chemotherapy, SIDCHM provided significantly better survival outcomes to patients with advanced or metastatic ESCC, with relative increases in median survival and PFS of 33.6% and 26.3%, respectively, with no obvious differences found in the incidence of adverse events (5). Undeniably, sintilimab may be an important option for immunotherapy in advanced or metastatic ESCC. However, the price of sintilimab is still high compared with chemotherapy, which may significantly increase healthcare expenditures, and previous economic analyses regarding the first-line treatment of advanced or metastatic ESCC with ICI have not been cost-effective from the Chinese healthcare system perspective (11, 25). Therefore, a cost-benefit analysis of sintilimab for advanced or metastatic ESCC is imperative.

Based on the ORIENT-15 trial (5), our economic analysis showed that compared with that of PLCHM, the ICER of SIDCHM as first-line therapy for advanced or metastatic ESCC in China was $23,458.08/QALY, and the probability of SIDCHM being cost-effective was 86% when the WTP threshold was set at $37,653/QALY. A major innovation in our study is the discovery of cost-effective first-line ICI therapeutic options for advanced or metastatic ESCC. The results of the subgroup analysis showed that most subgroups of patients preferred treatment with ADCHM owing to >50% probability of cost-effectiveness as compared to PLCHM, except for subgroups with programmed cell death ligand 1 expression (CPS)<1. A few pharmacoeconomic evaluations were conducted on sintilimab, with only three studies retrieved in PubMed, but their economic results were all cost-effective, which is consistent with our findings. Two economic studies using clinical data from the ORIENT-32 trial (26) demonstrated that compared with sorafenib, sintilimab plus bevacizumab may provide a cost-effective treatment for Chinese patients with unresectable hepatocellular carcinoma (14, 27). Rui et al. reported that sintilimab plus chemotherapy as a first-line therapeutic option for nonsquamous non-small-cell lung cancer of locally advanced or metastatic was more cost-effective in China in comparison with karilizumab plus chemotherapy (28). Therefore, to reduce the economic burden of medical treatment and provide more accessibility to Chinese patients, more attention should be paid to whether to use SIDCHM as a first-line therapeutic option for advanced or metastatic ESCC compared to other ICIs from the perspective of policymaking in China. In other words, the present results have a significant reference value for the Chinese healthcare system in developing first-line therapeutic options for advanced or metastatic ESCC. We believe very strongly that SIDCHM has also the potential to become a cost-effective therapeutic option for other cancers because of its price advantage and positive anti-tumor effect, which needs more economic research.

Inevitably, this study has some limitations. First, given the shortage of long-term survival data, we must consider using the Weibull survival model to make inferences about survival outside of the follow-up time, which may not provide an accurate reflection of real-world conditions. We will update our cost-effectiveness analysis when longer-term data on survival becomes available. Second, in the setting of disease progression, we chose to offer BSC to all patients due to the absence of relevant survival datasets for the enrolled patients, which may not accurately represent clinical practice at this time. We will analyze this further when relevant treatment costs and survival data for patients after progression are available. Third, in the model, we considered only the most common serious adverse events (SAEs) of level 3 and higher. The sensitivity analyses showed that the economic results were insensitive to SAE-associated. Fourth, although 7% of the patients in the ORIENT-15 trial received chemotherapy that was different from the preferred regimen in China, which may affect the economic evaluation in China, the results of the sensitivity analysis do not support the finding that this affected the economic results. Fifth, we had assumed that all patients chose 200 mg of sintilimab in the model, which is different from the real clinical situation. However, this would strengthen our economic results rather than change them because it raises the cost of the SIDCHM group. Sixth, changes in exchange rates affect direct medical costs, but the results of the one-factor sensitivity analysis indicate that healthcare costs do not change the results of the model when they change within the range we set. Finally, given the lack of relevant survival utility values for China, the utility values in this present study were obtained from other countries, which may result in model outcome bias. However, the results of the sensitivity analysis suggest that this does not affect the results of our economic evaluation. Despite these limitations, which cannot be ignored, this study is important as an economic reference for Chinese decision-makers to decide whether SIDCHM can be used as a first-line therapeutic option for advanced or metastatic ESCC.

## Conclusions

Current relevant guidelines for advanced or metastatic ESCC do not recommend SIDCHM as a first-line treatment option. However, the results of this study suggest that from the Chinese healthcare system perspective, SIDCHM is a cost-effective therapy choice for advanced or metastatic ESCC compared with conventional chemotherapy. Our findings will provide an important economic basis for the Chinese healthcare system to decide whether to use SIDCHM as a first-line treatment option for advanced or metastatic ESCC.

## Data availability statement

The original contributions presented in the study are included in the article/**Supplementary Material**. Further inquiries can be directed to the corresponding authors.

## Author contributions

Study design and supervision: MY, YHu, and ZC. Data analysis and interpretation: QW and WZ. Data collection: YHe and RC. Manuscript writing: MY. Final approval of the manuscript: All authors.

## Supplementary material

Supplementary Figure
**(A)** Survival curves for model and the ORIENT-15 trial in the SIDCHM group. **(B)** Survival curves for model and the ORIENT-15 trial in the PLCHM group. OS, overall survival; PFS, progression-free survival; PLCHM, placebo plus chemotherapy.
