# Supplementary figures and images for: Cost-effectiveness analysis of sintilimab plus chemotherapy for advanced or metastatic esophageal squamous cell carcinoma
Source: Front Oncol. 2022 Dec 8;12:986762. doi: 10.3389/fonc.2022.986762 (PMC9773135; doi:10.3389/fonc.2022.986762)

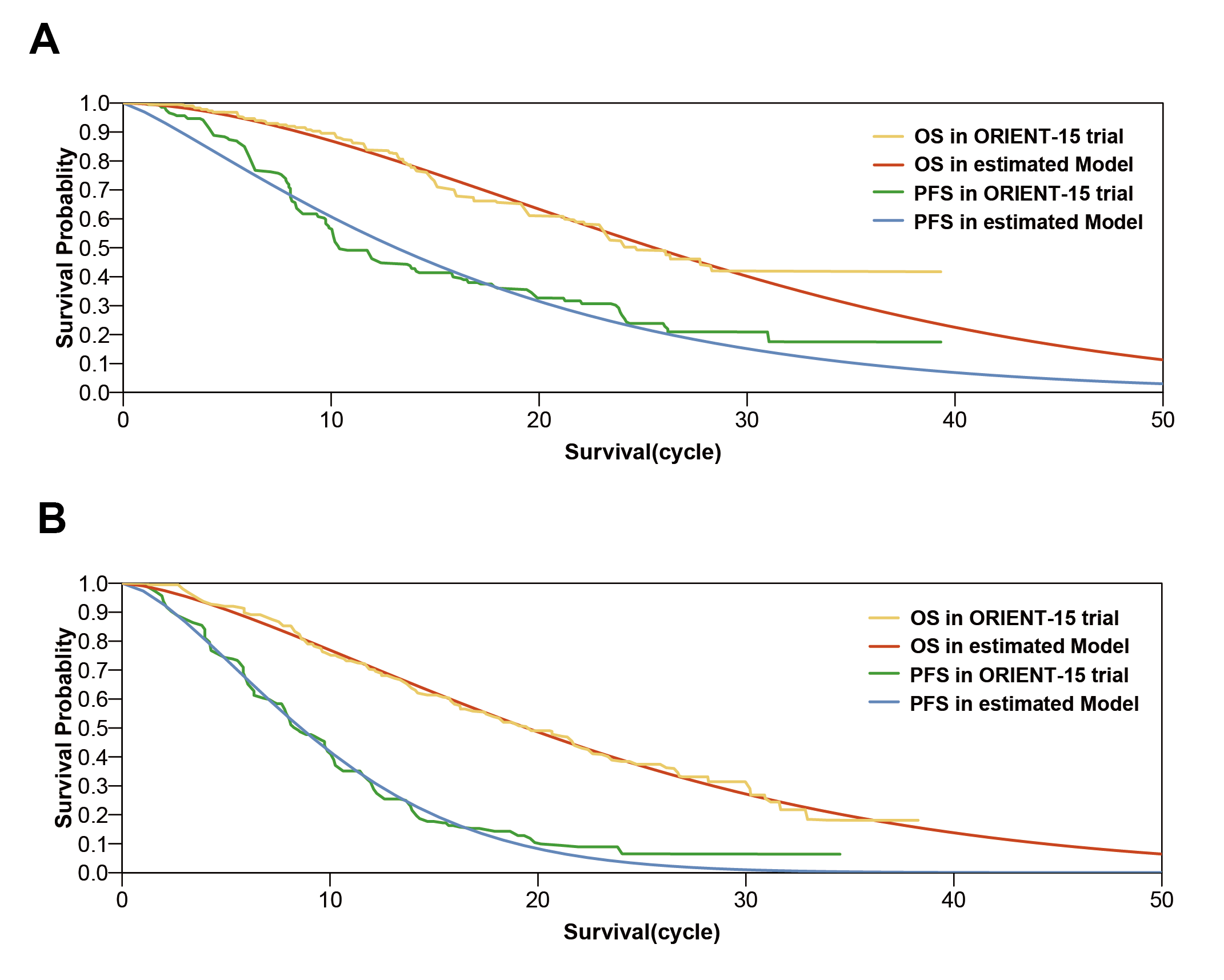

Supplement: Supplementary Figure — (A) Survival curves for model and the ORIENT-15 trial in the SIDCHM group. (B) Survival curves for model and the ORIENT-15 trial in the PLCHM group. OS, overall survival; PFS, progression-free survival; PLCHM, placebo plus chemotherapy. [file Image_1.tif]
